# 1127. Case Study. Multidisciplinary Approach to Long Thoracic Nerve Injury: A Case Report

**DOI:** 10.1093/jbcr/irag033.549

**Published:** 2026-04-06

**Authors:** Mihir S Kulkarni, Daniel Hernandez, Sai Ram Velamuri

**Affiliations:** University of South Florida Health Morsani College of Medicine, Tampa, FL; Tampa General Hospital, Tampa, FL; University of South Florida Health Morsani College of Medicine, Tampa, FL

## Abstract

**Patient Presentation (age range, injury details, relevant history):**

A 27-year-old male presented with 38% total body surface area deep partial- and full-thickness burns to the trunk and all extremities, sustained after contact with a loose electrical wire while seated in an electrical truck. His prior medical history was unremarkable. On admission, a fasciotomy of the right lower extremity was performed due to concern for compartment syndrome. Occupational therapy (OT) care began on hospital day two. Over the next four weeks, the patient underwent multiple surgical procedures while continuing rehabilitative therapy.

**Clinical Challenges:**

Electrical burn injuries, particularly high-voltage exposures, often cause deep tissue damage extending beyond the visible surface. Such injuries carry a risk of systemic complications, including cardiac, vascular, and neurologic sequelae that may appear in a delayed manner. Peripheral nerves are highly susceptible, leading to demyelination, axonal loss, or entrapment neuropathies, which can compromise long-term functional outcomes.

In this patient, after previously demonstrating full range of motion of the right shoulder, new-onset weakness of the serratus anterior was observed, with scapular winging two days later.

**Management Approach:**

The multidisciplinary team, including surgery and rehabilitation, was alerted. OT interventions focused on serratus anterior strengthening exercises aimed at restoring scapular stability and improving function.

**Outcomes:**

Despite intensive therapy, scapular winging persisted two weeks later. The patient was discharged to a local inpatient rehabilitation facility for ongoing burn care and functional therapy. Surgical intervention was not indicated at that time.

**Lessons Learned:**

This case illustrates a rare complication of long thoracic nerve injury following electrical burns, resulting in serratus anterior palsy. Neurologic injuries in the context of burns are uncommon but represent a significant rehabilitative challenge. Although surgical treatment was not required, a structured rehabilitation program is critical to optimize recovery and function.

**Applicability to Practice:**

To date, only three similar cases of delayed long thoracic nerve injury after electrical burn have been documented. This case adds to the limited literature on long-term neurologic complications associated with high-voltage burn injuries and underscores the importance of vigilance in rehabilitation planning.

